# Roles of microRNAs in Ovarian Cancer Tumorigenesis: Two Decades Later, What Have We Learned?

**DOI:** 10.3389/fonc.2020.01084

**Published:** 2020-07-21

**Authors:** Ali A. Alshamrani

**Affiliations:** Department of Pharmacology & Toxicology, College of Pharmacy, King Saud University, Riyadh, Saudi Arabia

**Keywords:** microRNA, ovarian cancer, proliferation, biomarkers, chemoresistance, diagnosis, prognosis, target genes

## Abstract

Ovarian cancer is one of the top gynecological malignancies that cause deaths among females in the United States. At the molecular level, significant progress has been made in our understanding of ovarian cancer development and progression. MicroRNAs (miRNAs) are short, single-stranded, highly conserved non-coding RNA molecules (19–25 nucleotides) that negatively regulate target genes post-transcriptionally. Over the last two decades, mounting evidence has demonstrated the aberrant expression of miRNAs in different human malignancies, including ovarian carcinomas. Deregulated miRNAs can have profound impacts on various cancer hallmarks by repressing tumor suppressor genes. This review will discuss up-to-date knowledge of how the aberrant expression of miRNAs and their targeted genes drives ovarian cancer initiation, proliferation, survival, and resistance to chemotherapies. Understanding the mechanisms by which these miRNAs affect these hallmarks should allow the development of novel therapeutic strategies to treat these lethal malignancies.

## Introduction

Ovarian cancer is the eighth leading cause of cancer-related deaths among females worldwide, with an estimated 295,414 new cases and 184,799 deaths as of 2018 (1). Even though the 5-year relative survival rate at stages I–II ranges between 75 and 92%, in the western world, for instance, the rate remains <30% for patients presenting at the clinic with advanced peritoneal dissemination and massive ascites (2). One of the main reasons for the high mortality rates is the lack of an efficient and sensitive method that can detect ovarian cancer at early stages. Current diagnostic approaches, including serum levels of CA125, pelvic examination, and transvaginal ultrasonography, have failed to detect this disease at the early stages (3).

Approximately 90% of primary malignant ovarian tumors arise from the ovarian surface epithelium (OSE). Morphologically, epithelial ovarian carcinomas are divided into four major types (serous, endometrioid, clear cell, and mucinous) (4). The heterogeneity of ovarian cancer is suggested to account for the high mortality rates caused by this disease (5). Ovarian carcinomas are also classified into low-grade and high-grade cancers based on their expression profiles of signature proteins or their characterized mutations, which define their aggressiveness and response to chemotherapies. At the molecular level, low-grade ovarian tumors characterized by several signature mutations, including PTEN, PIK3CA KRAS, BRAF, ERBB2, and ARID1A, whereas the more aggressive high-grade tumors are associated with TP53 mutations (6). In addition to the importance of these protein-coding genes, advances in genetics revealed that the mammalian genome transcribes thousands of short non-protein-coding RNAs that regulate various biological processes, among which are microRNAs (miRNAs). Extensive analysis of more than 1493 small RNA deep sequencing datasets estimated that the human genome transcribes about 1917 miRNA precursor sequences, generating 2654 mature miRNA sequences (7). MiRNAs are short, single-stranded, highly conserved non-coding RNA molecules (19–25 nucleotides) that negatively regulate the targeted protein-coding genes post-transcriptionally through binding with their 3′-UTR (untranslated region). Such binding facilitates the degradation of these genes' mRNAs or blocks their translation (8). The synthesis and processing of miRNAs are illustrated elsewhere (9). Briefly, miRNAs are first transcribed in the nucleus by the RNA polymerase II into long, double-stranded precursors called primary miRNA transcripts (pri-miRNAs), which are then processed by the nuclear RNase III microprocessor complex Drosha and DGCR8 to produce 60–70-nucleotide RNA hairpin-like intermediates called premature miRNAs (pre-miRNAs). The pre-miRNAs are then translocated primely to the cytoplasm by a RNA.GTP-bound protein, exportin-5, where the latter is cleaved by another RNase III enzyme, the Dicer complex, into 19–25-nucleotide RNAs (mature miRNAs). Mature miRNAs are then incorporated into a ribonucleoprotein complex known as the RNA-induced silencing complex (RISC), which, in collaboration with the Argonaute (AGO) protein, directs the miRNA complex to the targeted mRNA and facilitates its binding through perfect or imperfect base pairing. Since the miRNA binding to the 3′-UTR of the targeted mRNA does not require perfect complementarity, in addition to its ability to recognize strands as short as 2–8 nucleotides complementary to their 5′-seeding region, it has been estimated that one miRNA is capable of regulating the expression of several 100 genes and that one mRNA is regulated by multiple miRNAs.

Recent bioinformatic analysis has predicted that approximately two-thirds of all human genes are regulated by more than 1,000 miRNAs (10), which has prompted scientists in the last decade to investigate the roles of these miRNAs in many diseases, including cancer. Emerging evidence suggests that deregulated miRNAs play important regulatory roles in the initiation, progression, and dissemination of different types of cancer (11). It has become well-established that miRNAs can either be upregulated or downregulated in various human cancers, including ovarian cancer. Oncogenesis can be initiated either by the overexpression of oncogenic miRNAs, which downregulate tumor suppressor genes, or by the loss of specific tumor-suppressive miRNAs, which negatively regulate oncogenes (12). Since the first report establishing a connection between miRNAs and cancer (13), the importance of aberrant miRNA expression in different malignancies has been firmly established (47,009 PubMed hits in February 2020). Of these, 1,714 PubMed hits report diagnostic, prognostic, and therapeutic implications of miRNAs in ovarian cancer. This review summarizes the mechanisms by which miRNAs drive the transformation and tumorigenesis of different types of ovarian carcinomas. This work provides a deeper understanding of how miRNAs and their respective, experimentally verified target genes control different aspects of ovarian cancer pathogenesis.

## Aberrant Expression of miRNAs: Potential Diagnostic and Prognostic Biomarkers of Ovarian Cancer

### miRNA Expression Profiles in Ovarian Cancer Tissues

A substantial number of studies have investigated the differential expression profiles of miRNAs in the serum, plasma, exosomes in serum, ascites fluids, and tissues of ovarian cancer patients and normal counterparts to identify potential diagnostic and prognostic biomarkers. One of the first reports investigating the miRNA expression profiles in tissues obtained from ovarian cancer patients and normal subjects revealed signature expression patterns between the two groups. Among the commonly upregulated miRNAs regardless of the morphological histotypes reported in this study were miR-141and miR-200a-c, whereas miR-125b, miR-140, miR-145, and miR-199a were among the downregulated miRNAs in ovarian cancer tissues. This study also reported distinctive expression profiles of miRNAs within ovarian cancer tissues with different histopathologic features (serous, endometrioid, clear cell, and mucinous). For example, when compared with serous, endometrioid histotype seems to significantly upregulate miR-212 and miR-302b^*^, and downregulate miR-222 (14). The Lower number of samples of clear cell and mucinous carcinoma subtypes makes it harder to draw solid conclusions or safely distinguish between expression profiles. In another study, Zhang et al. have also assessed the alterations in the expression levels of miRNAs and their association with the malignant transformation of the OSE, the origin of EOC (15). This study compared the mature miRNA expression profiles of 18 EOC cell lines and four immortalized non-malignant human ovarian surface epithelium (IOSE) primary cell lines using multiple comprehensive miRNome techniques. Their results showed that 31 miRNAs were downregulated in the EOC lines compared with the IOSE cell lines, including the tumor suppressor miRNAs lethal-7 (let-7d) (16) and miR-127 (17). Analysis of primary human ovarian cancer specimens revealed that late-stage tumors significantly downregulated tumor suppressors miRNAs, miR-15a, miR-34a, and miR-34b, when compared with early-stage specimens. In an attempt to discriminate between primary and recurrent ovarian cancers, Laios et al. utilized TaqMan RT-PCR assay to quantify the miRNA expression profiles of primary and recurrent serous papillary adenocarcinomas. Their study revealed significant up and downregulation of miR-223 and miR-9, respectively, in recurrent tumors relative to the primary ones (18). Kaplan-Meier analysis of 20 patients with serous ovarian carcinomas revealed that higher expression of miR-18a, miR-93, and miR-200 family members (miR-141, miR-429 miR-200a-c), and lower expression of let-7b, and miR-199a were significantly correlated with decreased progression-free survival (PFS) and overall survival (OS) (19). The similarity between identified miRNAs in this study and the dysregulated miRNAs reported by Iorio et al. (14) further insinuate the importance of miRNAs as prognostic biomarkers. A few years later, Calura et al. confirmed the results demonstrated by previous studies when they analyzed the miRNA profiles for each EOC histotype at early stages to identify signature biomarkers for different ovarian carcinoma subtypes. This study demonstrated histotype-specific miRNA signatures for clear cell and mucinous ovarian cancer tissues. The clear cell histotype was characterized by high expression of miR-30a-5p and miR-30a-3p, whereas miR-192 and miR-194 were upregulated in the mucinous histotype (20).

A recent study examined the differentially expressed miRNAs in high-grade serous ovarian carcinoma (HGSC), clear cell ovarian carcinoma (CCC) and OSE. Both malignant subtypes mostly overexpressed miR-200 family members and miR-182-5p, whereas miR-383 was significantly underexpressed relative to OSE expressions. Higher expression of miR-509-3-5p, miR-509-5p, miR-509-3p, and miR-510 distinguished CCC from HGSC. Despite the relatively small number of subjects, higher miR-200c-3p expression was associated with poor PFS and OS in HGSC patients (21). More subtypes-oriented research is needed with a larger number of participants to validate signature miRNA biomarkers for the diagnosis and prognosis of ovarian cancer patients. The increasing number of reports that miRNAs expression profiles could be influenced by ethnicity and genetic backgrounds (22–24), urges for considering such a factor in the future studies aiming for identifying miRNA-based biomarkers for ovarian carcinomas. In addition to the two reasons pointed out above, the discrepancies of reported miRNA signature profiles in the tissues of ovarian cancer patients can be attributed to the heterogeneity of dissected tissue sample, the way these samples were processed, the number of miRNAs analyzed, the molecular assays used to detect these miRNAs, and the lack of standard methods for normalization (25). A more consistent, non-invasive, and less biased source of sampling is likely to produce more robust, reliable, and unified results that could help in the diagnosis, prognosis, and treatment of ovarian cancer patients.

### Circulatory miRNA Expression Profiles

**Figure 1:**
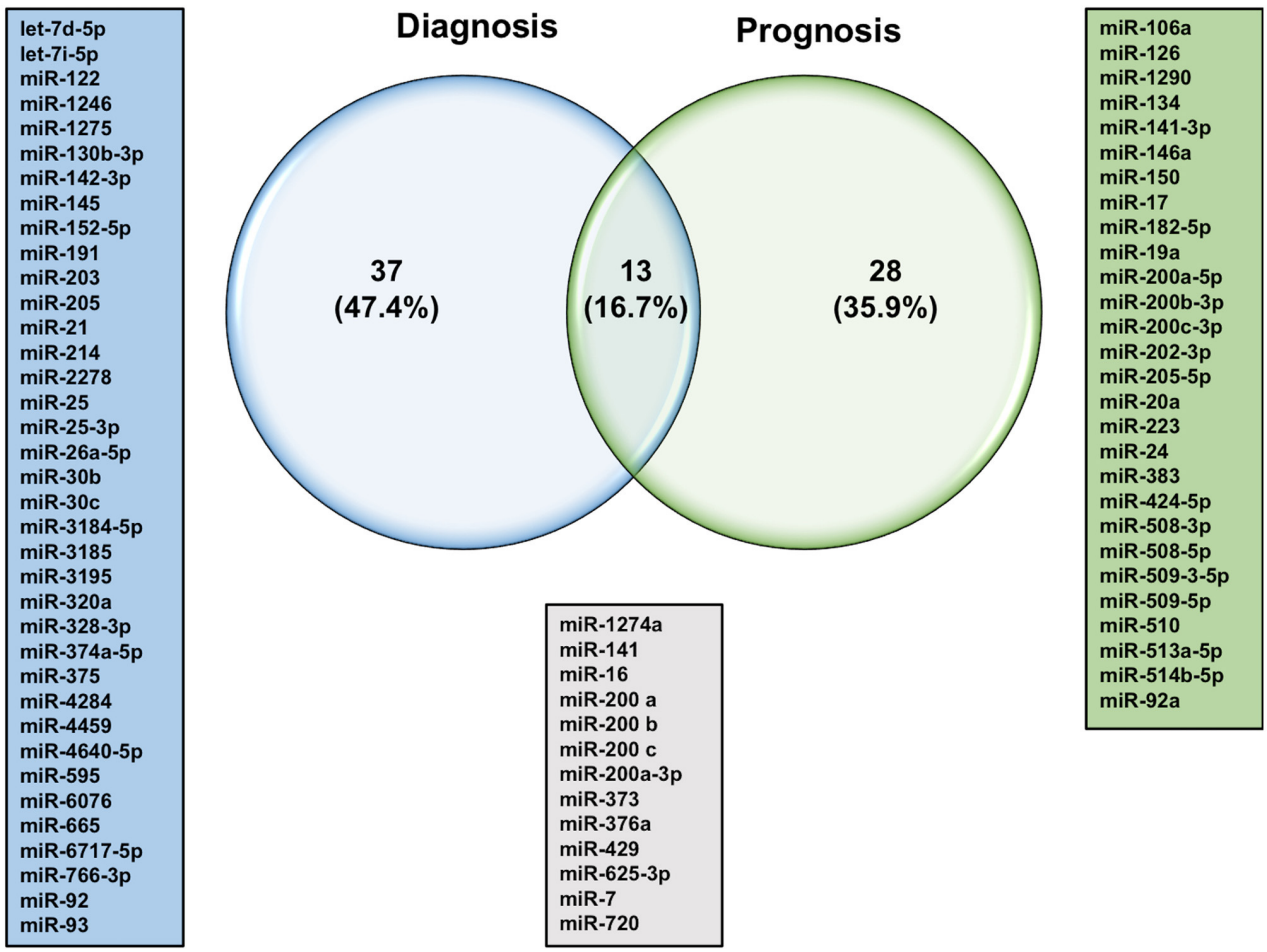
Venn diagram of potential diagnostic and prognostic miRNAs in ovarian cancer.

The quest for finding more reliable and distinctive diagnostic biomarkers for ovarian cancer, especially in the early stages, has been a priority of the scientific community for years (12). The differential expression of circulatory miRNAs has long been a more appealing approach for identifying clinical biomarkers for several reasons. miRNAs are found in the plasma and serum encapsulated in extracellular vesicles or bound with special lipid proteins, which make them resistant to RNase digestions (26). Several lines of evidence reported that these miRNAs have faster biogenesis and activation rates, and longer half-lives relative to mRNA and proteins, which make them suitable for detection at early stages (diagnostic), during progression or treatment selection (prognostic) (27, 28). One of the pitfalls of using single circulatory miRNA is the lack of specificity. For instance, several miRNAs such as miR-21-5p, miR-155-5p, and miR-210-3p have shown different expression patterns in different types of cancers, including lung, colorectal, and breast cancer (29). Therefore, the is an urgent need to employ advances in cancer genetics to identify specific miRNA signature patterns instead of stand-alone miRNA for the clinical diagnosis of ovarian cancer. Yokoi et al. have developed a miRNA-based detection method using comprehensive expression profiles of 10 serum-isolated miRNAs specific to ovarian tumors and helped discriminate ovarian tumors from other types of solid tumors and non-cancer cells (miR-320a, miR-665, miR-3184-5p, miR-6717-5p, miR-4459, miR-6076, miR-3195, miR-1275, miR-3185, and miR-4640-5p) (30). More specifically, a recent study identified miR-508-3p as a master regulator of the mesenchymal subtype of ovarian cancer and a promising prognostic biomarker that could help to diagnose this lethal disease in its early stages (31). Most of the studies that have investigated the expression profiles of miRNAs in the circulation of ovarian cancer patients are summarized in Table 1. Only studies that used samples from more than 20 ovarian cancer patients were included in this table. Some of these miRNAs showed consistent expression patterns in the circulation of ovarian cancer patients. For example, miR-25 was reported by two independent studies to be downregulated in the serum samples of ovarian cancer patients (40, 41). Pathway enrichment analysis revealed that miR-25-3p targets the oncogenic Toll-like receptor-4 (TLR-4)/MD88 pathway, which could result in overexpression of the latter (40). On the other hand, the vast majority of studies report significant upregulation of the miR-200 family members in the circulation of ovarian cancer patients except for one study that showed only miR-200c family member to be upregulated (47). The variability in sample processing and quantification techniques utilized in this study might count for undetectable levels of other family members, including miR-141, miR-200a, and miR-200b. This study differs from others that showed upregulated miR-200 family in two little details, the lower number of qRT-PCR cycles and the absence of the cDNA preamplification step. In addition, results from Table 1 were used to determine which miRNAs could serve as diagnostic and/or prognostic biomarkers. As shown in Figure 1, of the 50 dysregulated miRNAs that are reported here in ovarian cancer patients relative to healthy individuals, only 13 miRNAs (16.7%) demonstrated the ability to serve as diagnostic and prognostic biomarkers in ovarian cancer patients, among which are the miR-200 family members. Further extensive studies with a higher number of subjects are needed to investigate the expression patterns of those 13 miRNAs at different stages of the disease, their ability to distinguish different ovarian cancer subtypes, and their utilization as predictors of the disease outcomes and biomarkers for disease relapse.

**Table 1:** MicroRNA expression profiles in different ovarian cancer subtypes in relation to normal controls.

**Study design (*****n*****)**		**Specimen**	**Decreased miRNAs**	**Elevated miRNAs**	**References**
**Tumor histotype**	**Controls**				
SOC (50)	HC (10) BOA (10)	Serum exosomes	–	miR-21, miR-141, miR-200 family (a,b,c), miR-203, miR-205, miR-214	(32)
SOC (17), Mixed EOC & MOC (5), COC (6)	HC (15)	Serum	miR-99b, miR-127, miR-155	miR-21, miR-29a, miR-92, miR-93, miR-126	(33)
SOC (20), EOC (2), Unspecified solid tumors (2)	HC (15)	Whole blood	miR-181a*, miR-342-3p, miR-450-5p	miR-30c1*	(34)
HGSOC (28)	HC (28)	Serum	–	miR-200 family (a,b,c)	(35)
SOC (21), COC (7), EOC (6), Mixed EOC & COC (1)	HC (20) EM (33)	Plasma	–	SOC: miR-16, miR-191, miR-4284 EOC: miR-16, miR-21, miR-191, miR-195	(36)
SOC (42)	HC (23) BOA (36)	Plasma	**>**10-fold: miR-16, miR-17, miR-19b, miR-20a, miR-24, miR-92a, miR-106a, miR-126, miR-146a, miR-150, miR-223 **<**10-fold: miR-30a-5p, miR-30b, miR-30c, miR-106b, miR-191, miR-193a-5p, miR-320, miR-328	miR-625-3p, miR-720, miR-1274a	(37)
SOC (16), COC (14), EOC (15) MOC (12), Unspecified (17)	HC (50)	Serum	–	miR-141, miR-200c	(38)
SOC (18), COC (17), EOC (24) MOC (12), Unspecified (13)	HC (135) BOA (51)	Serum	miR-145	–	(39)
SOC (25)	BOA (25)	Serum	let-7i-5p, miR-25-3p, miR-122, miR-152-5p	–	(40)
EOC (180)	HC (66)	Serum	miR-25, miR-93	miR-7, miR-429	(41)
(163) mainly Serous	HC (32) BOA (20)	Serum exosomes	–	miR-200 family (a,b,c), miR-373	(42)
Unspecified (32)	HC (10)	Serum	–	miR-376a	(43)
HGSOC (168)	HC (65)	Serum	–	miR-595, miR-1246, miR-2278	(44)
SOC (112), COC (19), EOC (13) MOC (11)	HC (63) BOA (43)	Serum	–	miR-26a-5p, miR-130b-3p, miR-142-3p, let-7d-5p, miR-200a-3p, miR-328-3p, miR-374a-5p, miR-766-3p	(45)
HGSOC (56)	HC (30)	Serum	–	miR-375	(46)
HGSOC (39) Non HGSOC (9)	BOA (10) BOT (10)	Serum exosomes	miR-141, miR-200a, miR-200b	miR-93, miR-145, miR-200c	(47)

*SOC, Serous Ovarian Cancer; HGSOC, High-Grade Serous Ovarian Carcinoma; COC, Clear Cell Ovarian Cancer; EOC, Endometrioid Ovarian Cancer; MOC, Mucinous Ovarian Cancer; BOA, Benign Ovarian Adenoma; HC, Healthy Control; EM, Endometriosis; BOT, Borderline Ovarian Tumor*.

## MicroRNAs Regulate the Initiation of Ovarian Carcinomas

The initiation of cancer is a complex multi-step process that requires inactivation of tumor suppressor genes and the activation of oncogenes. The regulatory roles of miRNAs in the initiation of ovarian cancer comprise a rich area of research that has yet to be further explored. Substantial evidence has linked dysregulation in miRNAs expression with the early stages of ovarian cancer transformation. In humans, for example, the consensus let-7 family of tumor suppressor miRNAs has been suggested by numerous studies to be downregulated in several types of cancer, including breast, lung, stomach, colon, and ovarian cancer (48, 49). Among the reported target genes of the let-7 family are the early embryonic genes HMGA2 and IMP-1 (50) as well as oncogenic NRAS (16). The embryonic high mobility group AT-hook 2 (HMGA2), a direct target of let-7 (51–53), is a non-histone, nuclear-binding, oncofetal protein that has been shown to be overexpressed in embryonic tissue and many malignancies, including high-grade ovarian carcinomas (54, 55). In addition, HMGA2 functions as a crucial regulator of cell growth and differentiation (56). Shell et al. have provided the first direct association in human cancer between the downregulation of let-7 and the upregulation of HMGA2. Their study revealed that ovarian cancer patients with high HMGA2 and low let-7 expression in their cancer tissues had lower survival rates than patients with low HMGA2 and high let-7 expression (57). In another study, Wu et al. reported that overexpressing HMGA2 initiated the malignant transformation of OSE cell lines *in vitro* and *in vivo* and induced more aggressive tumor cell growth (55). Taken together, this evidence suggests that the downregulation of the let-7 family of miRNAs may serve a critical function in the malignant transformation of OSE into EOC. However, further loss and gain of function experiments are needed to demonstrate the direct impact of the tumor suppressor let-7 on the initiation and malignant transformation of ovarian cancer.

Previous studies have reported that ovarian cancer tissues had significantly lower miR-199a expression profiles relative to normal ovarian cells, which has been associated with poor PFS and OS in patients with serous ovarian carcinoma (14, 19). CD44^+^/CD177^+^ cells are ovarian cancer-initiating stem cells (CIC), which are known to be highly proliferative and resistant to chemotherapeutics, with low differentiation capabilities (58–60). Findings from a recent study by Cheng et al. revealed that miR-199a targets CD44 in these cells resulting in significant suppression of cell cycle, proliferation capacity, and invasiveness *in vitro* and tumor growth *in vivo* (61). These data suggest that the deregulation of miR-199a expression may serve as a prerequisite for the initiation of ovarian cancer.

During the differentiation process, tumor-initiating stem cells lose their stemness and acquire the characteristics of fast-growing, chemoresistant mature cancer cells. For instance, twist-related protein 1 (Twist1)-mediated upregulation of miR-199a/214 has been shown to be essential for the transformation of ovarian cancer stem cells (Type I/CD44^+^) into mature, fast-dividing cancer cells (Type II/CD44^−^) (62). At the molecular level, the upregulation of miR-214 and miR-199a resulted in a reduction of the tumor suppressors PTEN and IKKβ, which led to the activation of the AKT and NFκB pathways, respectively. All these independent findings further suggest that miRNAs can serve as vital regulators of the initiation of ovarian cancer. Further studies are needed to better elucidate the direct regulatory roles of miRNAs on the stemness, growth, and differentiation of ovarian cancer-initiating cells. Other critical components of the tumor initiation and progression process, including enhanced proliferative capabilities, evading immunocompetent cells, uncontrolled cell division, and maintaining survival (63), and how miRNAs regulate each step will be discussed in more detail later.

## MicroRNAs Regulate Proliferation, Cell Cycle, and Survivability of Ovarian Carcinomas

**Figure 2:**
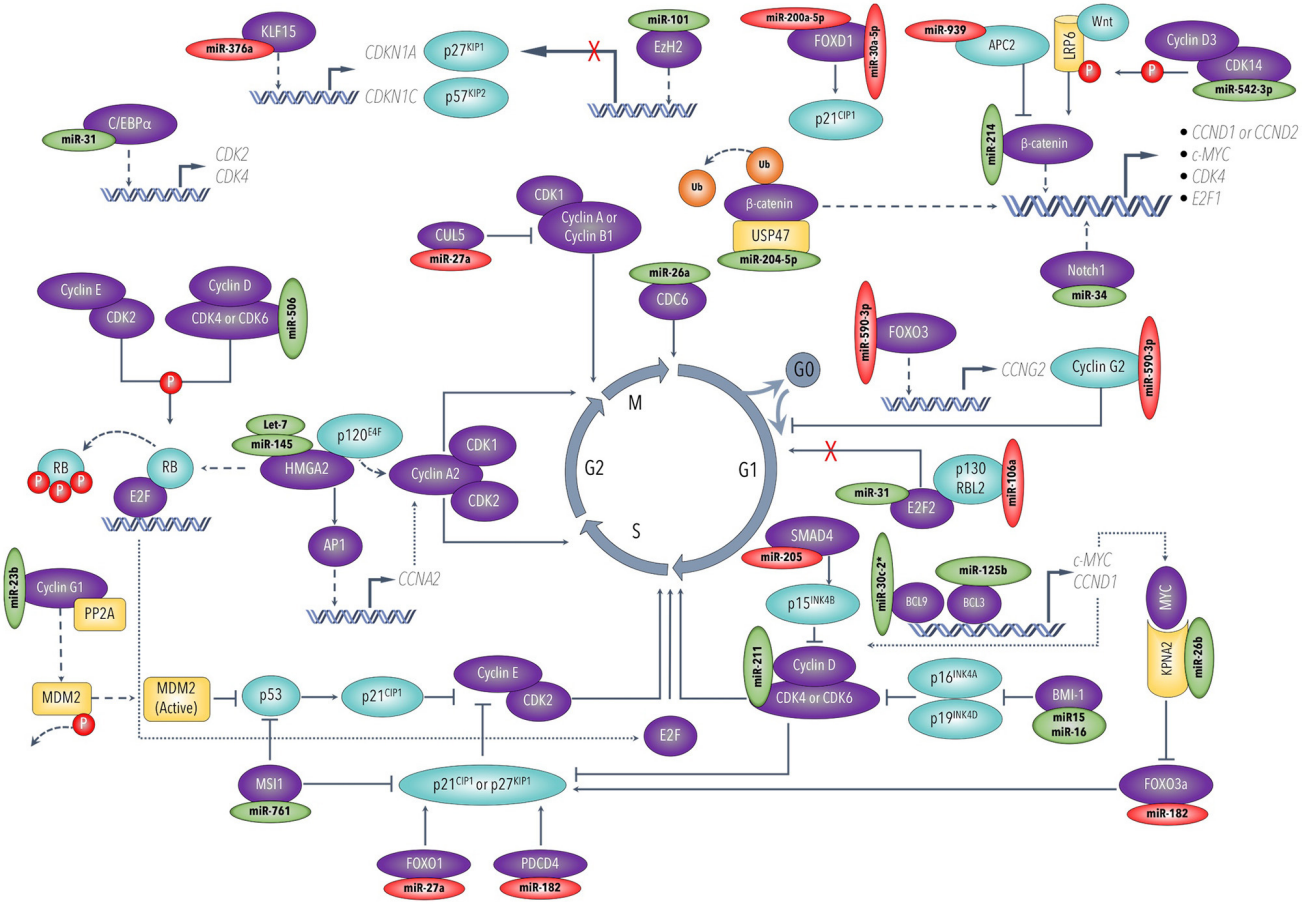
Cell cycle progression and major regulatory miRNAs in ovarian cancer. This figure was drawn based on the information from Liu et al. (183), Shi et al. (190), Qie et al. (221), and Liu et al. (224).

The ability of cancer cells to maintain active proliferation and survive in harsh and continuously changing environments are two of the most critical hallmarks of neoplastic diseases (64). Cell cycle progression is a multifaced process heavily guarded by several growth factors and suppressors, including cyclins and their protein kinases and inhibitors (65). These proteins regulate the cell quiescence (G0 phase), cell cycle re-entrance (G0-G1), and cell cycle progression through different phases (G1, S, G2, M) (Figure 2) Over the years, an increasing number of studies has aimed to understand the mechanisms of how miRNAs influence tumor suppressors and oncogenes to regulate cell proliferation and cell cycle progression pathways in ovarian cancers.

The cyclin-dependent kinases (CDKs), CDK4/6 and CDK14, have emerged as important targets for miRNAs that have decreased expression in ovarian cancer, namely, miR-506 (66), miR-211 (67), miR-542-3p (68). Ectopic expression of these tumor-suppressive miRNAs inhibits ovarian cancer cell proliferation and cell cycle progression through different phases by engaging multiple downstream pathways. Mechanistically, the abundant CDK4/6 can promote cell cycle progression via two major mechanisms, as illustrated in Figure 2. First, CDK4/6 phosphorylates and releases the retinoblastoma (Rb) tumor suppressor protein and thus enables E2F transcription factors to upregulate downstream targets involved in cell cycle transition from G1 into S phase. Second, CDK4/6 reactivates cyclin E-CDK2 kinase by sequestering CDK inhibitors p21^CIP1^ and p27^KIP1^ (69). In another study, miR15a/ miR-16 were reported to be downregulated in ovarian cell lines and primary ovarian tissues. The resultant overexpression of their target proto-oncogene Bmi-1 promoted cell cycle progression by suppressing CDK4/6 repressors p16^INK4a^ and p19^INK4d^, and thus drive the cell cycle forward at G1/S phase (70). CDK14, on the other hand, is reported to activate several cell cycle-controlling targets, downstream of Wnt/β-catenin signaling pathway, by phosphorylating the co-receptor lipoprotein receptor-related protein 6 (LRP6) (71).

In addition to the increased expression of CDKs, miRNAs can also modulate the expression of several cell cycle-regulating cyclins directly or indirectly. For instance, Salem et al. recently reported that miR-590-3p enhanced ovarian cancer cell proliferation by directly binding and suppressing the cell cycle repressor, cyclin G2 (72). Furthermore, ovarian cancer cells perhaps “strategically” keep the tumor suppressor miR-23b at lower expression to maintain proliferative status by increasing the expression of its oncogenic target CCNG1 (cyclin G1) to sufficient levels to activate Mdm2 and ultimately override inhibitory effects of the wild-type p53 (73, 74). Other cyclins have been shown to be modulated indirectly when their upstream controlling proteins are directly affected by miRNAs. For example, several lines of evidence reported that overexpressed HMGA2 conversely correlates with the expression levels of its regulators let-7 and miR-145, in the ovarian cancer tissues and cell lines (53, 57, 75). As shown in Figure 2, HMGA2 can drive the cancer cell cycle forward by multiple mechanisms (56). Briefly, HMGA2 directly binds and suppresses p120^E4F^ transcriptional repressor, thus inducing cyclin A2 expression, a cell cycle regulator at the S and G2/M phases. HMGA2 can also induce cyclin A2 transcription by upregulating its upstream regulator activator protein-1 (AP1). In addition, HMGA2 facilitates the cell cycle G1-S phase transition by releasing E2F1 transcription factors from the suppressor protein Rb. More recently, miR-27a-mediated inhibition of the E3 ubiquitin ligase Cullin-5 (CUL5) might result in maintaining high levels of cyclin A and B1, which are required for G2-M phase transition and cell proliferation (76).

In addition to the direct impacts of miRNAs on the cell cycle checkpoint regulators, other signaling targets upstream of the cell cycle and proliferation have been shown to be manipulated by miRNAs in ovarian cancer. For example, the class O of forkhead box transcription factors (FOXO) and the extracellular signal transducers, SMADs, exhibit suppressive roles by inducing cell cycle arrest (65). Mechanistically, FOXO1 inhibits cell proliferation, apparently by inducing the expression of the CDK inhibitor p21^Cip1^ (77). A recent study revealed that oncogenic miR-27a could stimulate ovarian cancer cell proliferation by suppressing the expression of FOXO1 (78). The tumor suppressor FOXO3 can also induce cell cycle arrest by increasing the transcription of CDK inhibitors p21^CIP1^ and p27^KIP1^ (79, 80), and cyclin G2 (72). Multiple studies suggest that ovarian cancer cells maintain lower levels of FOXO3 presumably by upregulating several oncogenic miRNAs, including miR-182 (81) and miR-590-3p (72).

**Figure 3:**
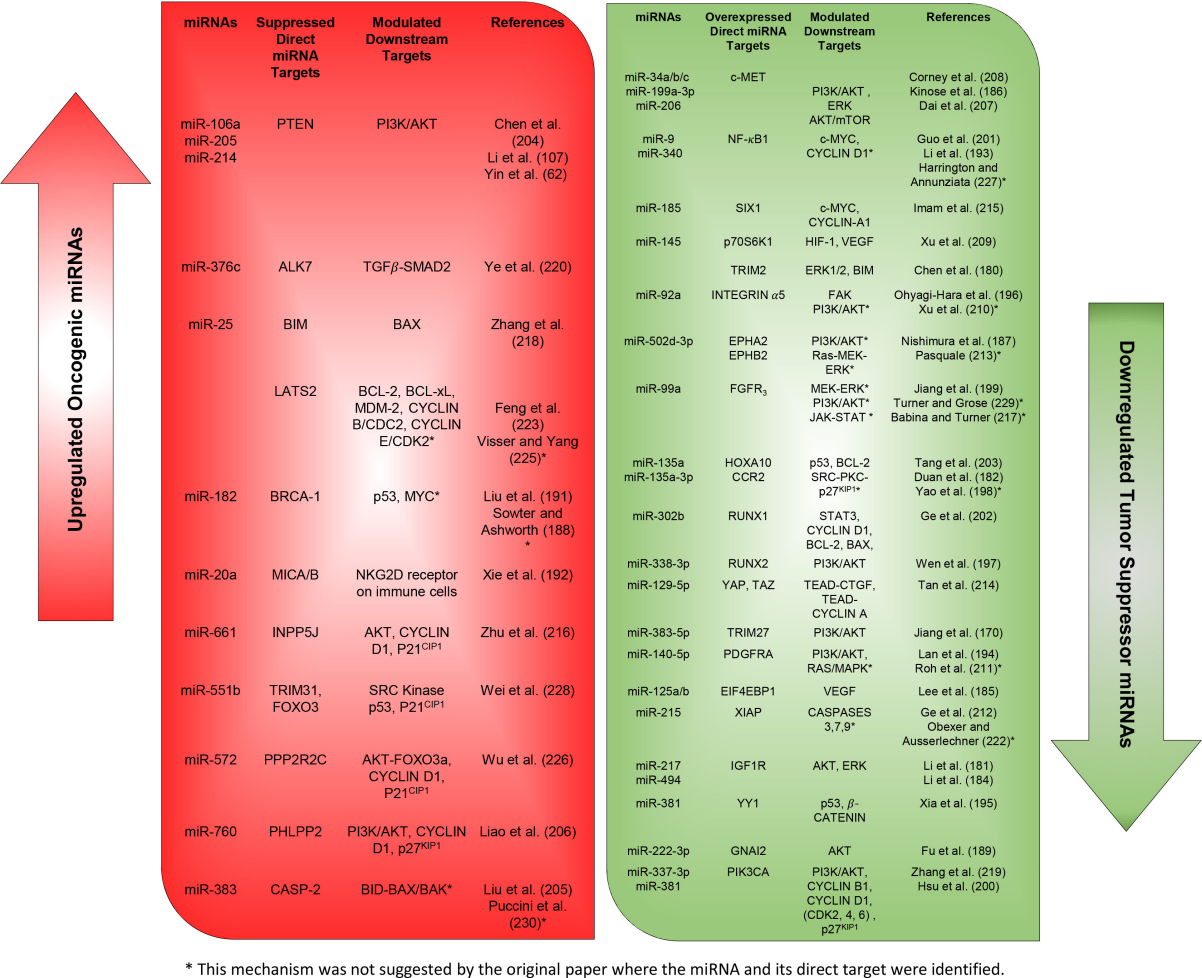
Expressions of oncogenic and tumor suppressor miRNAs and their reported downstream targets associated with cellular proliferation and survival in ovarian cancer.

More studies that have investigated different miRNAs, their validated targets, and the downstream modulated targets that play essential roles in ovarian cancer proliferation and survival capabilities are listed in detail in Figures 2, 3. In addition, Table 2 summarizes more possible mechanisms by which miRNAs regulate the cell cycle progression and proliferation in ovarian cancer cells based on the reported targets. Since some of these mechanisms were not demonstrated in ovarian cancer cells *per se*, more studies are encouraged to validate that what has been shown in other cancers can be applied to ovarian cancer.

**Table 2:** Mechanisms by which microRNAs enhance ovarian cancer cell cycle and proliferation.

**References**	**miRNA**	**miRNA expression in ovarian cancer cells**	**Target gene**	**Possible mechanisms**
Creighton et al. (82)	miR-31	Down	C/EBPα E2F2	Activated C/EBPα upregulates cdk2/4 and activates the AKT signaling pathway, which stimulates cell cycle progression, presumably by facilitating transitions through the S and G2/M phases (83). Activated E2F2 stimulates cell cycle progression, as demonstrated above.
Jia et al. (84)	miR-30c-2*	Down	BCL9	The resultant overexpression of the oncogene BCL9 increases cell proliferation through upregulating cyclin D1 expression.
Semaan et al. (85)	miR-101	Down	EzH2	The overexpressed enhancer of EzH2 represses the tumor suppressor protein P21^Cip1^, thus promoting G1/S phase transition.
Guan et al. (86)	miR-125b	Down	BCL3	Similar to BCL9, the overexpressed proto-oncogene BCL3 can form a complex with the NF-κB p50 and p52 isoforms. This complex activates the cyclin D1 promoter, increasing the rate of cell proliferation.
Cheng et al. (61) Sun et al. (87)	miR-199a miR-3129	Down Down	CD44	CD44 is a transmembrane glycoprotein that is overexpressed in ovarian cancer and has been shown to promote cell proliferation by signaling through the MAPK pathway.
Liu et al. (88)	miR-182	Up	PDCD4	Downregulation of the tumor suppressor protein (programmed cell death 4, PDCD4) enhances cell proliferation by either inhibiting the cell cycle repressors p21^Cip1^ and p27^kip1^ (89) or upregulating cyclin D1 (90).
Liu et al. (91)	miR-106a	Up in HGSOC	p130 (RBL2)	As a member of the Rb family of proteins, RBL2 typically forms a complex with E2F2, preventing cell cycle progression and inducing cell cycle arrest at the G0/G1 phases. As a result of the miR-106a-mediated downregulation of RBL2 in ovarian cancer, these cells become highly proliferative due to the absence of this complex.
Lenkala et al. (92)	miR-22	Up	MXI-1	MXI-1 is a member of the Mad family of transcription factors that directly represses the transcriptional activity of the c-Myc oncogene promoter, resulting in the suppression of cell proliferation (93). Inhibition of the MXI-1 transcription factor could allow c-Myc to activate downstream proliferation-related genes (94).
Li et al. (95)	miR-210	Up in hypoxic EOC	PTPN1	One of the mechanisms by which EOC adapts to hypoxic conditions is through inhibiting the pro-apoptotic PTPN1 (96). When present, PTPN1 induces cell death by different mechanisms, including activation of caspases 8/9 and potentiation of inositol-requiring enzyme (IRE1) receptor signaling during endoplasmic reticulum stress, in addition to the downregulation of several pro-survival receptor-tyrosine kinases such as EGFR.
Ying et al. (97)	miR-939	Up	APC2	APC2 is a tumor suppressor that negatively regulates the Wnt/β-catenin signaling pathway. Downregulation of this tumor suppressor stimulates the Wnt/β-catenin pathway, which promotes cellular proliferation by upregulating and activating downstream genes, such as Cyclin-D1, c-Myc, and T cell factor (TCF) proteins.
Lin et al. (98)	miR-26b	Down	KPNA2	The resultant overexpression of the nuclear transport protein KPNA2 can positively regulate cell cycle transition at the S/G1 phase through upregulation of and enhancing the transcriptional activity of c-Myc, Akt, and cdk regulator cyclin D1 and by downregulation of cyclin-dependent kinase inhibitors p21^Cip1^ and p27^Kip1^ and FOXO3a activity (79).
Sun et al. (99)	miR-26a	Down	CDC6	Cdc6 is an oncogene that plays a crucial role in regulating the cell cycle transition between the G1/S phase.
Xiaohong et al. (100)	miR-203	Up	PDHB	Downregulation of PDHB allows cancer cells to maintain active proliferative status by enhancing cytosolic glycolysis and lactate production in the surrounding tumor microenvironment (101).
Bi et al. (102)	miR-127-3p	Down	BAG5	Upregulated BAG5 binds the tumor-promoting protein mutant p53, which prevents E3 ubiquitin ligases-mediated degradation and the ubiquitination of the latter. Accumulation of mutant p53 has recently been reported to enhance tumor cell proliferation (103).
Yang et al. (43)	miR-376a	Up	KLF15	Several studies have reported the reciprocal relationship between KLF15 expression and cell cycle regulators, such as E2F1 and cyclin-cdk inhibitors p21^Cip1^/p57^kip2^, in different types of cancer (104–106).
Li et al. (107)	miR-205	Up	SMAD4	Downregulation of the intracellular effector protein SMAD4 prevents TGF-β-mediated cell cycle arrest, presumably by repressing downstream p21^Cip1^ and p27^Kip1^ (108).
Chen et al. (109)	miR-193a-3p	Down	GRB7	GRB7 is a multidomain adaptor protein that transduces EGFR proliferative and anti-apoptotic signals by MAPK/ERK activation, Bcl-2 upregulation, and, Bax repression (110).
Wang et al. (111)	miR-30a-5p, miR-200a-5p	Up	FOXD1	The resultant downregulation of the tumor suppressor FOXD1 drives the cell cycle forward by maintaining low expression and activity of the cell cycle-inhibitor, p21^Cip1^, in a p53-independent manner.
Sheng et al. (112)	miR-206	Down	KIF2A	KIF2A is a non-motile microtubule depolymerase that plays a crucial role in depolarizing microtubule ends during cell division.
Jia et al. (113)	miR-34	Down	Notch 1	Several proliferation-related genes respond positively to Notch signaling, including cyclin-dependent kinase inhibitors p21^Cip1^ and p27^Kip1^, cyclin D1, and oncogenic c-Myc (114).
Hu et al. (115)	miR-204-5p	Down	USP47	USP47 is a member of the deubiquitinating enzyme family and is suggested to regulate cell proliferation through stabilizing β-catenin in Wnt signaling (116).

## Roles of Micrornas in Ovarian Cancer Chemosensitivity and Resistance

### miRNA-200 Family

The miR-200 family consists of five miRNA members (miR-141, miR-200a, miR-200b, miR-200c, and miR-429), whose expressions are reported to be significantly deregulated in different ovarian cancer tissues and cell lines (14, 19, 32, 35). Recent studies have linked miR-200 family members with the responsiveness of ovarian cancer patients to chemotherapeutic drugs. For example, p38α MAPK was previously shown to play an important role in reducing tumorigenesis by regulating cell proliferation, survival, and stress response (117, 118). Mateescu et al. identified p38α MAPK as a direct target for two of the most highly expressed miRNAs in ovarian cancer tissues, miR-141 and miR-200a (119). Both miR-141 and miR-200a increased paclitaxel sensitivity in a ROS-dependent manner by repressing p38α MAPK *in vivo*. More recently, a study by Liu et al. also reported that ectopic expression of miR-200a sensitized the ovarian cancer cell line OVCAR-3 to paclitaxel (120). MiR-200c is one of the most comprehensively investigated miRNAs in ovarian cancer. Although the majority of studies have shown that miR-200c is highly expressed in the tumor tissues as well as serum of ovarian cancer patients (32, 35), other studies have demonstrated that miR-200c was significantly downregulated in many advanced, poorly differentiated ovarian adenocarcinomas (121, 122). It seems that what controls the expression of these miRNAs are the disease stage, the type of specimen, and whether or not these cells have been exposed to treatment. Cochrane et al. reported that exogenous overexpression of miR-200c in different ovarian cancer cell lines resulted in a dramatic reduction in the expression of class III β-tubulin (TUBB3), a highly prevalent mechanism of resistance to microtubule-binding chemotherapies in many solid tumors (123–125). A study by Leskela et al. confirmed the previous findings by demonstrating that highly resistant ovarian tumors had high levels of the TUBB3 protein and significant downregulation in the expression of miR-141, miR-429 and miR-200c (126). Studies at the other end of the spectrum revealed that high expression of miR-141 and miR-200c was strongly correlated with ovarian cancer resistance to platinum-based therapy. Ectopic overexpression of miR-141 induced cisplatin resistance in ovarian cancer cells by directly suppressing the expression of the tumor suppressor Kelch-like ECH-associated protein 1 (KEAP1) and subsequently activating its downstream NF-κB pathway (127).

Epithelial-mesenchymal transition (EMT) is another biological process by which cancer cells confer resistance to chemotherapy (128). Increasing evidence suggests that the expression of key EMT-associated genes is directly regulated by several members of the miR-200 family. For instance, Zinc-finger E-box binding homeobox 1, ZEB1 (also known as δEF1), and zinc finger E-box-binding homeobox 2, ZEB2 (also known as SIP1), the upregulation of which facilitate EMT and tumor metastasis by downregulating E-cadherin, were identified as direct targets of all five members of the miR-200 family. Intriguingly, inhibition of these microRNAs was sufficient to induce EMT and reduce adhesion by upregulating E-cadherin transcriptional repressors, ZEB1, and/or SIP1 (121, 129). In agreement with these studies, two independent research groups have demonstrated that resorting to the expression of miR-429 and the other miR-200 family members induced mesenchymal-to-epithelial transition (MET), reverted the EMT phenotype, and sensitized different ovarian cancer cells to Taxol and platinum-based chemotherapies (130, 131). Furthermore, a study by Chen et al. demonstrated that miR-200c was significantly downregulated in the more aggressive and chemoresistant phenotype CD117^+^/CD44^+^ ovarian cancer stem cells compared with the CD117^−^/CD44^−^ cells. Ectopic overexpression of miR-200c in the CD117^+^/CD44^+^ cells inhibited EMT and induced tumor suppression by directly repressing ZEB1 and Vimentin, which resulted in a subsequent upregulation of the E-cadherin expression (122). Collectively, these observations support the notion that the miR-200 family of microRNAs influences the sensitivity of ovarian cancer cells to conventional chemotherapies through multiple mechanisms, including, but not limited to, interference with cellular microtubule assembly, regulating NF-κB signaling, and the EMT/MET processes.

### miRNA-214/199a Cluster

The upregulation of miR-214 in various human malignancies, including ovarian cancers, and its roles in chemosensitivity and resistance have been extensively studied and documented. One of the earliest works by Yang et al. demonstrated a significant upregulation of miR-214 expression (8.63-fold) in late-stage, high-grade tumors compared to the normal ovarian tissues. Mechanistic experiments revealed that miR-214 negatively regulated PTEN by binding to its 3′-UTR, leading to the repression of its translation and subsequent activation of its downstream Akt signaling cascade. The resulting activation of the AKT pathway by miR-214 promoted ovarian cancer cell survival and cisplatin resistance. Furthermore, miR-214 knockdown abrogated cisplatin resistance in the cisplatin-resistant cells (132). Since then, several studies have reported a reciprocal relationship between miR-214 expression and the degree of resistance to multiple therapies in numerous malignancies, including pancreatic (133) and gastric cancers (134, 135). Wang et al. have recently demonstrated that exosome-containing anti-miR-214 resensitized cisplatin-resistant gastric cancer cells *in vitro* and *in vivo*, serving as a promising treatment approach for refractory gastric cancer (136).

MiR-199a^*^ (also known as miR-199a-5p) is another downregulated miRNA in ovarian cancer tissues relative to normal ovarian counterparts and has been implicated as playing an important role in ovarian cancer sensitivity to chemotherapeutics. In 2008, a study conducted by Chen et al. identified for the first time the functional role of miR-199a^*^ as a direct inhibitor of IKKβ expression, a kinase that phosphorylates IκB proteins, causing their proteasomal degradation and subsequent nuclear translocation of NF-κB. They reported that Type I/MyD88 positive EOC cells had the capacity to develop resistance to cytotoxic drugs such as paclitaxel and TNF-α due to lower miR-199a^*^ expression and higher IKKβ expression (137). In another study, HIF-1α and HIF-2α were identified as direct targets for miR-199a^*^. High expression of these two hypoxia-responsive factors and low expression of miR-199a^*^ mediated ovarian cancer cell resistance to carboplatin. This observation was validated when exogenous overexpression of miR-199a^*^ in the EOC cell line (A2780) disrupted cells' peritoneal seeding and sensitized them to carboplatin treatment *in vivo* (138). CD44^+^/CD117^+^ cells are ovarian cancer-initiating cells (CIC) that are characterized by their highly proliferative capabilities and their resistance to chemotherapies (58, 59). Cheng et al. found that miR-199a significantly enhanced the chemosensitivity of ovarian CICs to Adriamycin, cisplatin, and paclitaxel, and reduced the mRNA expression of the multidrug resistance gene ABCG2 (61). Most recently, Liu et al. have reported that miR-199b^*^ (also known as miR-199b-5p) was significantly downregulated in cisplatin-resistant ovarian cancer cells relative to cisplatin-sensitive cells. They found that the loss of miR-199b-5p activated the JAG1-mediated Notch1 signaling cascade, a reported chemoresistance and survival-regulating pathway in different types of human malignancies (139, 140). Restoration of miR-199b-5p expression resensitized the ovarian cancer cells to cisplatin-induced cytotoxicity both *in vitro* and *in vivo*. Findings from these studies further insinuate the diversity of targets of these miRNAs and their pivotal roles in modulating cellular response to chemotherapies.

### Let-7 Family

**Figure 4:**
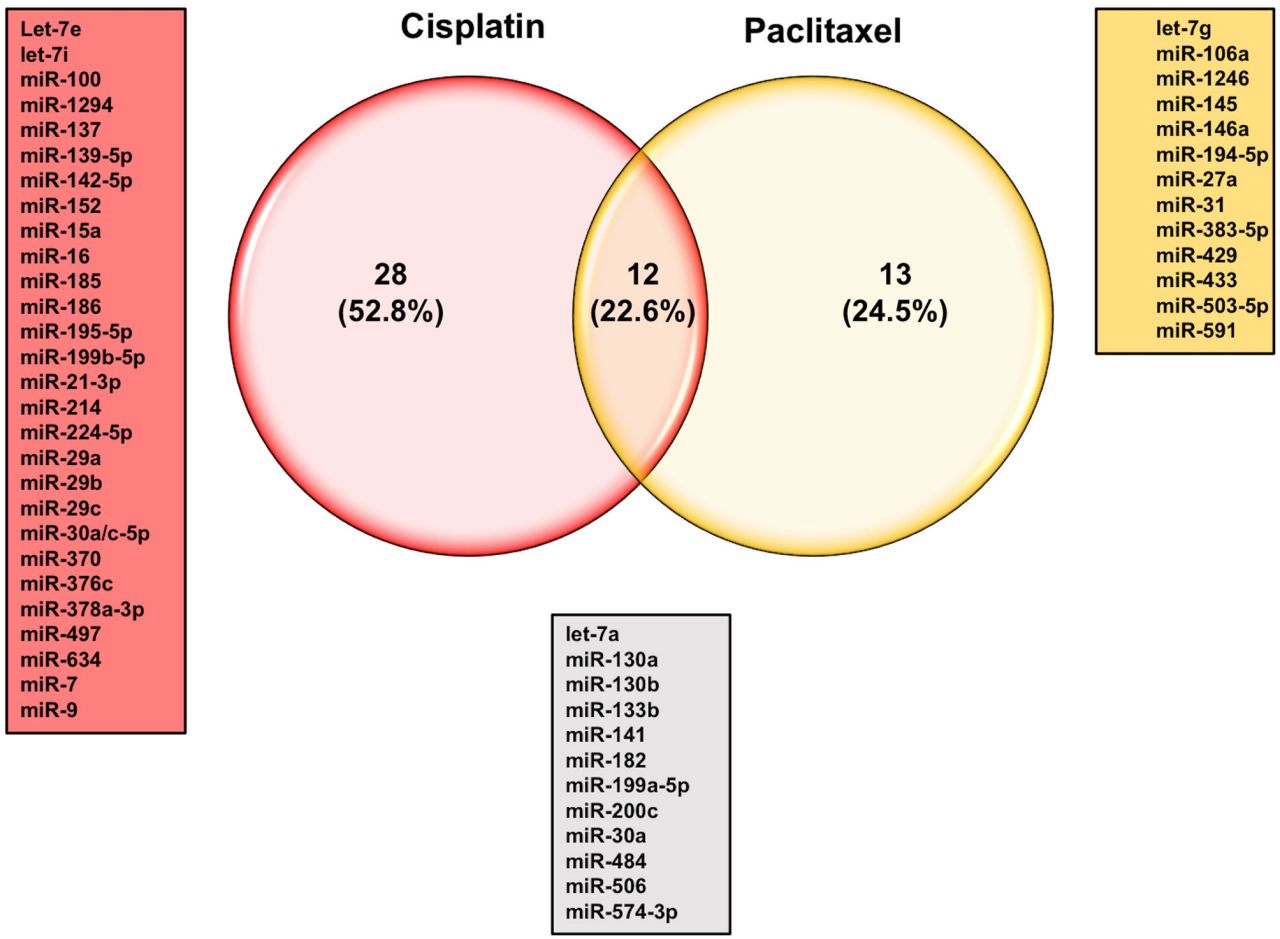
Venn diagram of reported miRNAs that strongly modulate ovarian cancer sensitivity to chemotherapeutic agents.

**Figure 5:**
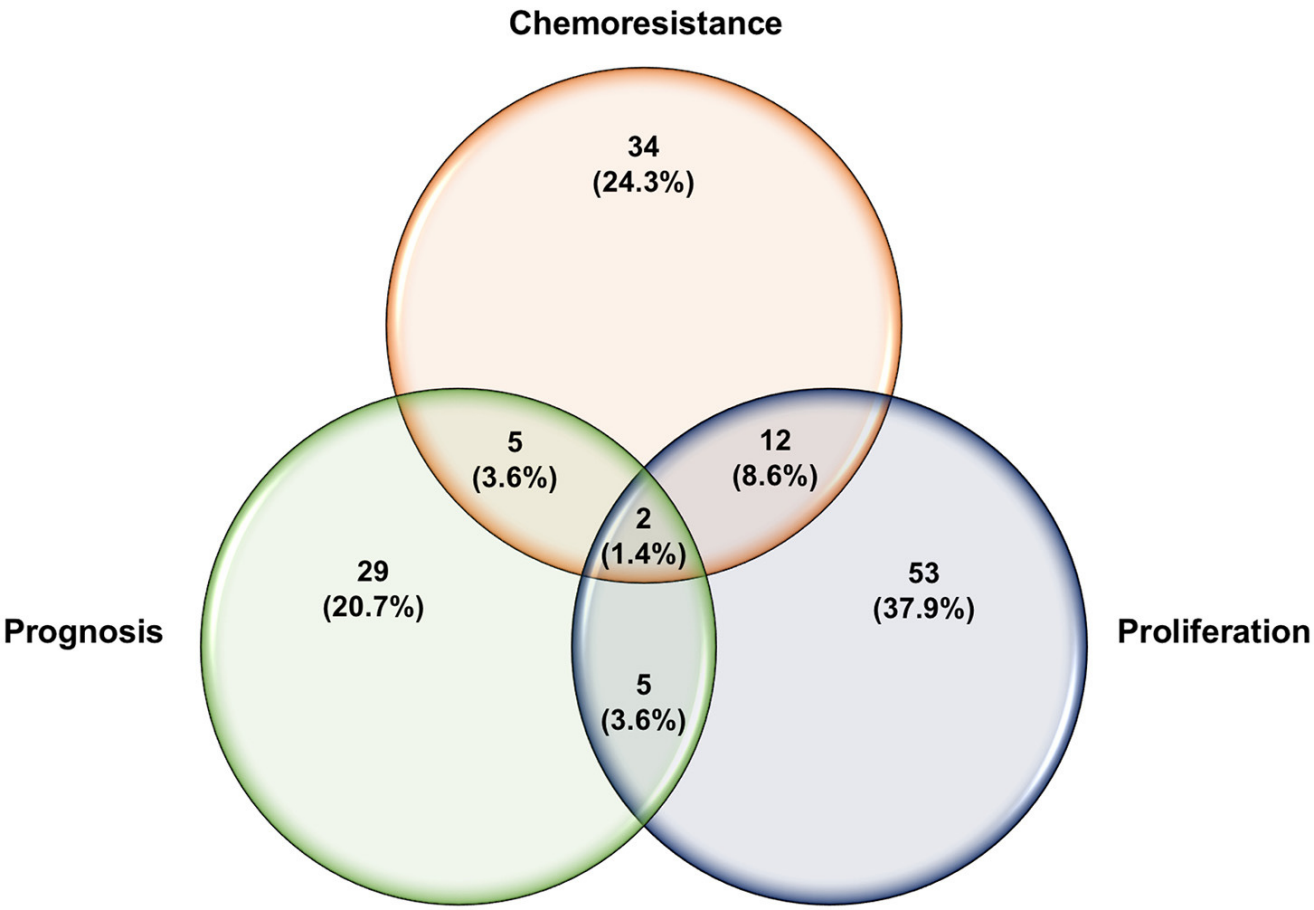
Venn diagram of overlapping miRNAs reported to regulate different aspects of ovarian carcinoma.

The let-7 (lethal-7) family of miRNAs consists of 13 human homologs that are located on nine different chromosomes (48, 141). Several studies have reported that the expression profiles of the let-7 family members were significantly reduced in various human cancer tissues and cell lines, including ovarian cancer (49–53, 57, 142). In addition to the roles of let-7 family members in the initiation and progression of ovarian cancer as mentioned above, studies have also shown a strong correlation between dysregulation in let-7 expression and the development of resistance to chemotherapeutic drugs. For example, Yang et al. have demonstrated that let-7i expression was significantly reduced in the tumor tissue samples derived from chemotherapy-resistant ovarian cancer patients. Loss-of-function (let-7i inhibition) and gain-of-function (let-7i overexpression) studies revealed that ovarian cancer resistance to cisplatin was significantly increased in the absence of let-7i, whereas ectopic expression of let-7i resensitized cells to treatment (142). Similarly, Boyerinas et al. reported that the sensitivity of multidrug resistant ovarian cancer cells to microtubule-targeting drugs (Taxanes) was increased by overexpressing let-7g in those cells. This effect was mediated by repressing IMP-1, which in turn causes destabilization of multidrug resistance (MDR1) at the mRNA and protein level, thereby increasing the sensitivity of the multidrug-resistant ovarian cancer cell to Taxanes but not carboplatin, a non-MDR1 substrate (143). Let-7e is another member of the let-7 family that has been reported recently to play an important role in the development of cisplatin resistance in ovarian cancer cells. Findings by Cai et al. demonstrated that let-7e expression was significantly downregulated in cisplatin-resistant human EOC cells compared to parental ones. Exogenous overexpression of let-7e resensitized the former to cisplatin by reducing the expression of cisplatin-resistant related proteins EZH2 and cyclin D1 (144). Deregulation of some members of the let-7 family of miRNAs have also been suggested to serve as potential biomarkers to predict the responsiveness of ovarian cancer patients to different chemotherapeutic agents. For instance, Lu et al. reported that ovarian cancer patients with higher let-7a expression did not respond well to paclitaxel/platinum combination therapy and had worse overall survival rates compared to patients with low let-7a expression (145). More recently, analysis of human EOC tissue specimens revealed that the tumor suppressors let-7g and let-7d-5p were strongly associated with acquired resistance to cisplatin (146, 147). Other individual miRNAs, their identified targets, and examples of the mechanisms by which these miRNAs could modulate the responsiveness of ovarian cancer cells to chemotherapy are listed in Table 3. Approximately 53% of the miRNAs reported in this review modulate ovarian cancer responsiveness to cisplatin, whereas ~25% play a significant role in cell resistance against taxanes-based therapy. As shown in Figure 4, members of the let-7 family, miR-214/199a cluster, and miR-200 family were among the 12 miRNAs that appear to be critical regulators of chemoresistance to cisplatin and paclitaxel. Furthermore, five chemoresistance-associated miRNAs in ovarian cancer patients (miR-141, miR-146a, miR-200c, miR-429, and miR-7) have been suggested to serve as novel prognostic biomarkers. Interestingly, only two miRNAs (miR-106a and miR-16) showed the ability to modulate proliferation and chemosensitivity of ovarian cancer cells and to function as prognosis indicators in ovarian cancer patients (Figure 5).

**Table 3:** Examples of the mechanisms of miRNA-mediated resistance in ovarian cancer.

**References**	**miRNA**	**Expression in resistant cells**	**Target gene**	**Suggested mechanism**
Xu et al. (148)	miR-497	Down	mTOR/P70S6K1	The resultant activation of mTOR/P70S6K1 positively affects several downstream effectors to regulate cell growth, proliferation, and survival (149).
Chen et al. (150)	miR-133b	Down	GST-π	GSTs have the ability to detoxify cytostatic drugs (151). Therefore, chemotherapy-resistant ovarian cancer cells might develop resistance by upregulating the miR-133b-target gene GST-π.
van Jaarsveld et al. (152)	miR-634	Down	CCND1, GRB2, ERK2, and RSK2	Upregulation and activation of these MAPK pathway components produce undesirable effects on several fundamental cellular processes, including cell cycle progression and inhibition of apoptosis (153).
Dwivedi et al. (154)	miR-15a miR-16	Down	BMI1	Bmi1, a member of the Polycomb Repressor Complex 1, is a crucial regulator of the self-renewal and malignant transformation of many cancers (see Table 2).
Cui et al. (155)	miR-146a	Down	SOD2	Resistant cells have shown higher levels of the antioxidant SOD2, which scavenge any reactive species produced by the chemotherapy and thus resist death (156).
Guo et al. (157)	miR-100	Down	mTOR and PLK1	Cells are able to develop resistance by enhancing the functions of the mitotic regulator gene Polo-like kinase 1 (Plk1) (158), in addition to the other oncogenic roles of mTOR (see above).
Zhu et al. (159)	miR-186	Down	Twist1	Twist1 can confer chemoresistance in different cancers by regulating several downstream signaling proteins, including mTOR and Bcl2. In addition, Twist1 binds and activates ABC transporters, mediates EMT-induced resistance, and positively regulates PI3K/AKT pathway (160).
Han et al. (161)	miR-30a/c-5p	Down	Snail	DNMT1-mediated silencing of miR-30a/c-5p upregulates E-cadherin-transcriptional repressor Snail, thereby promoting EMT-mediated resistance to cisplatin.
Vera et al. (162)	miR-7	Down	MAFG	Recent evidence suggests that MAFG confers antioxidant activity against free radicals produced as a result of cisplatin treatment, which might be the mechanism behind MAFG-induced cisplatin resistance (163).
Jiang et al. (164)	miR-139-5p	Down	C-Jun	The resultant overexpression of the proto-oncogene c-Jun promotes the expression of the anti-apoptotic protein Bcl-_XL_, thereby suppressing the apoptotic death of these cells.
Kanlikilicer et al. (165)	miR-1246	Up	Cav1	Upregulated miR-1246 potentiates resistance toward paclitaxel through the downregulation of Cav1, a protein that directly binds and inhibits the kinase activity of the platelet-derived growth factor receptor PDGFRβ, which correlates with induction of the p-glycoprotein (MDR1) levels.
Chen et al. (166)	miR-139-5p	Down	RNF2	Evidence suggests that ovarian cancer cells can develop resistance by upregulating the ring finger protein 2, RNF2, an E3 ligase that targets the tumor suppressor p53 for degradation, thereby inhibiting cellular apoptosis (167).
Zhang et al. (168)	miR-1294	Down	IGF1R	See Table 2
Xu et al. (169)	miR-378a-3p	Down	MAPK1/GRB2	See above
Jiang et al. (170)	miR-383-5p	Down	TRIM27	See above
Sun et al. (171)	miR-137	Down	EZH2	EZH2 functions as a transcriptional suppressor or a transcriptional co-activator through epigenetic regulation of histone methylation. Evidence has shown that the upregulation of EZH2 strongly correlates with cisplatin resistance (172, 173).
Park and Kim (174)	miR-503-5p	Down	CD97	CD97 is a member of the epidermal growth factor (EGF)-seven transmembrane family that signals through Janus-activated kinase 2(JAK2), a signal transducer and activator of transcription 3 (STAT3), to induce paclitaxel resistance in ovarian carcinoma.
Nakamura et al. (175)	miR-194-5p	Down	MDM2	Upregulation of the E3 ubiquitin ligase, MDM2, blocks the transcriptional activity of several tumor suppressor genes, including p53 and p21.
Li et al. (176)	miR-142-5p	Down	XIAP	See Table 2.
Zhang et al. (177)	miR-574-3p	Down	EGFR	Cells might confer resistance toward chemotherapies by activating several signaling cascades involved in proliferation and survival that are downstream of the oncogenic EGFR (178).
Dai et al. (179)	miR-195-5p	Down	PSAT1	PSAT1-promotes inactivating phosphorylation of GSK3β, allowing the accumulation and nuclear translocation of β-catenin, which might help in conferring resistance by upregulating the stress-related gene, HIF-1α.

## Concluding Remarks

The treatment of patients with ovarian cancer faces multiple challenges ranging from late diagnosis, due to the lack of reliable markers, to the development of resistance to current therapeutics owing to the heterogeneity of the disease. Over the last 20 years, substantial progress has been made in our understanding of how miRNAs regulate different cancer hallmarks and how we can utilize such knowledge to improve the diagnosis and prognosis of cancer patients. However, several challenges could affect the confidence in utilizing miRNAs as diagnostic and prognostic biomarkers, including diversified sampling methods, detection techniques, gender, and ethnicity or genetic background. More studies are needed with a larger number of patients to investigate the impact of each of the above factors on the identified miRNA signatures. Research on how miRNAs regulate the pathology of ovarian cancer is still in its infancy, representing <4% of the total published reports. The bulk of these studies deal with expression profiling, identification of target genes, determination of affected cellular pathways, resistance to conventional chemotherapeutics, and their utilization as therapeutic tools. As demonstrated in this review, overexpressing tumor suppressor miRNAs, using synthetic miRNAs mimics, or inhibiting the activity of oncogenic miRNAs, using anti-miRNA oligonucleotides, have a strong potential to serve as a therapeutic approach for the treatment of ovarian cancer.

## Author Contributions

AA herby confirm sole responsibility for all information submitted in this manuscript and also declare my 100% contribution in this manuscript.

## Conflict of Interest

The author declares that the research was conducted in the absence of any commercial or financial relationships that could be construed as a potential conflict of interest.

## Abbreviations

ALK7: activin receptor-like kinase-7
APC2: adenomatosis polyposis coli 2
ARID1A: AT-rich interaction domain 1A
BAG5: Bcl-2-associated athanogene 5
Bax: Bcl-2-associated X protein
BCL3: B-cell lymphoma 3
BCL9: B-cell CLL/lymphoma 9
Bim: Bcl-2 homologous 3
Bmi-1: polycomb ring finger
C/EBPα: CCAAT enhancer binding protein alpha
Cav1: caveolin-1
CCND1: cyclin D1
CCR2: C-C motif chemokine receptor 2
CDC6: cell division cycle 6
CTGF: connective tissue growth factor
CUL5: cullin5
E2F2: E2F transcription factor 2
EIF4EBP1: eukaryotic translation initiation factor 4E binding protein 1
EphA2: EPH receptor A2
EphB2: EPH receptor B2
EzH2: enhancer of zeste 2 polycomb repressive complex 2 subunit
FAK: Focal adhesion kinase
FGFR3: fibroblast growth factor receptor 3
FOXD1: forkhead box D1
FOXO1: forkhead box O1
FOXO3: forkhead box O3
GNAI2: G protein alpha inhibiting activity polypeptide 2
GRB7: growth factor receptor bound protein 7
GSK3β: glycogen synthase kinase 3 beta
GST-π: glutathione S-transferase pi 1
HIF-1α: hypoxia-inducible factor 1 alpha
HMGA2: high mobility group AT-hook 2
HOXA10: homeobox A10
IGF1R: insulin like growth factor 1 receptor
INPP5J: inositol polyphosphate-5-phosphatase J
KIF2A: kinesin family member 2A
KLF15: Kruppel like factor 15
KPNA2: karyopherin subunit alpha 2
LATS2: large tumor suppressor kinase 2
LRP6: LDL receptor-related protein 6
MAFG: MAF bZIP transcription factor G
MAPK1: mitogen-activated protein kinase 1
MICA/B: MHC class I polypeptide-related sequences A/B
MSI1: Musashi RNA-binding protein1
mTOR: mechanistic target of rapamycin kinase
MXI-1: MAX-interacting protein 1
NF-κB1: nuclear factor kappa B subunit 1
NKG2D: killer cell lectin like receptor K1
OLFM4: olfactomedin 4
p130 (RBL2): retinoblastoma transcriptional corepressor like 2
p70S6K1: 70kDa ribosomal S6 kinases
PAK3: p21 (RAC1) activated kinase 3
PDCD4: programmed cell death 4
PDGFRA: platelet-derived growth factor receptor alpha
PDHB: pyruvate dehydrogenase E1 subunit beta
PHLPP2: PH domain and leucine-rich repeat protein phosphatase 2
PIK3CA: phosphatidylinositol-4,5-bisphosphate 3-kinase catalytic subunit alpha
PPP2R2C: protein phosphatase 2 regulatory subunit B gamma
PSAT1: phosphoserine aminotransferase 1
PTEN: phosphatase and tensin homolog
PTPN1: protein tyrosine phosphatase non-receptor type 1
RSK2: ribosomal protein S6 kinase A3
RUNX1/2: runt-related transcription factor 1/2
SEMA4D: semaphorin 4D
Six1: SIX homeobox 1
SOD2: superoxide dismutase 2
STAT3: signal transducers and activators of transcription3
TAZ: tafazzin
TEAD: TEA domain
TGF-β: transforming growth factor β
TP53: tumor protein p53
TRIM2: tripartite motif containing 2
USP47: ubiquitin specific peptidase 47
VEGF: vascular endothelial growth factor
XIAP: X-linked inhibitor of apoptosis
YAP: Yes-associated Protein.
